# Draft genome sequences data of four *Salmonella enterica* subsp. *enterica* serovar Dublin archival strains originating from animals in Poland, 1956 – 1957

**DOI:** 10.1016/j.dib.2022.108721

**Published:** 2022-11-03

**Authors:** Milena Skóra, Renata Kwit, Magdalena Zając, Marta Pietruk, Magdalena Skarżyńska, Ewelina Skrzypiec, Katarzyna Tłuścik, Anna Lalak, Dariusz Wasyl

**Affiliations:** a Department of Microbiology, National Veterinary Research Institute, Pulawy, Poland; b Department of Omic Analyses, National Veterinary Research Institute, Pulawy, Poland

**Keywords:** *Salmonella* Dublin, Cattle, Fox, Whole-genome sequencing, Pathogen

## Abstract

*Salmonella enterica* subsp. *enterica* serovar Dublin (*S*. Dublin) is a zoonotic pathogen causing infections in animals, especially in cattle. In this study, we report draft genome sequences of four *S*. Dublin isolated between 1956 and 1957 from cattle and fox in Poland.

Whole genome sequencing was performed on the Illumina platform and the data is available at National Center for Biotechnology Information under the BioProject accession number PRJNA865912. In order to better understand the genetic basis of epidemiology of *S*. Dublin infection, the obtained sequences were analyzed using the tools which are available at Center of Genomic Epidemiology (https://www.genomicepidemiology.org/) including core genome multilocus sequence typing (cgMLST) and core genome single nucleotide polymorphisms (cgSNPs).

**Specifications Table**

**Table:** 

Subject	Biological sciences
Specific subject area	Microbiology: Bacteriology
Type of data	Genome sequence data, table, figure
How the data were acquired	Whole genome sequencing: Illumina MiSeq, Quality control: FastQC v0.11.5, Trimming sequences: Trimmomatic v0.36, Merge trimmed reads: BBMerge from bbtools software suite, Assembly: SPAdes v3.9.0, Assembly statistics: QUAST, Genome annotation: PGAP, Genome analysis: tools from Center of Genomic Epidemiology (CGE), Phylogenetic analysis: MEGA 6, Sequences visualization: Proksee server
Data format	Raw, filtered and assembled genome sequences
Description of data collection	Four lyophilized strains of *Salmonella* Dublin were revived, streaked onto Xylose Lysine Deoxycholate (XLD) medium and passaged onto nutrient agar. Genomic DNA was isolated from pure nutrient agar culture with the Maxwell RSC cultured cells DNA kit (Promega) sequenced with the Illumina Miseq platform.
Data source location	• Institution: National Veterinary Research Institute (PIWet) • City/Town/Region: Pulawy • Country: Poland • Latitude and longitude (and GPS coordinates, if possible) for collected samples/data: PIW15: 52.73371238 N 15.23034418 E PIW16, PIW19, PIW21: 54.46904475 N 17.04150334 E
Data accessibility	Assembled sequences and raw reads have been deposited in GenBank under the BioProject accession number PRJNA865912 and BioSample accession number: PIW 15 (SAMN30076191), PIW 16 (SAMN30076192), PIW 19 (SAMN30076193), PIW 21 (SAMN30076194).

## Value of the Data

• The draft genome data may be useful for estimating the degree of genetic diversity of *Salmonella* Dublin strains.
• The scientists could use these genome data for comparative genome analysis and also identify evolutionary changes among *Salmonella* Dublin.
• The draft genome data allow better understanding of *Salmonella* epidemiology.

## 1 Objective

The incidence of *S*. Dublin infection in cattle is a major problem from both animal and human health perspective. Whole genome sequencing allows accurate genome analysis of pathogens, invasiveness and pathogenicity mechanisms. This study describes the *S*. Dublin sequences genome to better understand the evolutionary changes that have occurred over dozens of years.

## 2 Data Description

*Salmonella enterica* serovar Dublin (*S*. Dublin) is one of the host-specific serovars adapted to cattle. Infections are detected in both calves and adult animals and can cause substantial losses in livestock production. Symptoms of salmonellosis include diarrhea, fever, loss of appetite, in pregnant cows abortion may occur [1]. *S*. Dublin was confirmed also in foxes and other fur-bearing animals [2] and can spread to different species as a result of interspecies transmission [3]. Human infections are caused by the consumption of food contaminated e.g. milk, dairy products [4], and after contact with infected animals [5]. Here, we present draft genome sequences of four isolates *S.* Dublin from cattle and fox isolated in the National Veterinary Research Institute (PIWet) between 1956 and 1957 and lyophilized between 1958 and 1960 and stored under refrigerated conditions until current use. *S*. Dublin genomes varied from 4,875,403 to 4,881,137 bp, and had between 31 and 37 contigs with an average GC content of 52.1%. More information about the described sequences was included in Table 1.

**Table 1:** Genome characteristics of *S*. Dublin sequences.

Isolate	PIW 15	PIW 16	PIW 19	PIW 21
Species	*Salmonella enterica*	*Salmonella enterica*	*Salmonella enterica*	*Salmonella enterica*
Serovar	Dublin	Dublin	Dublin	Dublin
Isolation date	1956	1956	1957	1956
Lyophilization date	1958	1960	1958	1958
Host	cattle	cattle (calf)	cattle	fox
Sample type	feces	internal organs	internal organs	internal organs
Genome size (bp)	4,875,403	4,876,577	4,881,137	4,878,038
No. of contigs	31	34	36	37
Total no. of reads	1,641,274	1,545,018	1,570,976	1,062,786
Overall read coverage (x fold)	101	95	97	66
%GC	52.1	52.1	52.1	52.1
N_50_ (bp)	679 274	679 108	560 267	480 263
MLST type	ST 10	ST 10	ST 10	ST 10
cgMLST	219,058	279,553	279,536	12,682
Plasmid replicon (identity%)	IncFII(S) 97.71, IncX1 98.66	IncFII(S) 97.71, IncX1 98.66	IncFII(S) 97.71, IncX1 98.66	Col(pHAD28) 91.15, IncFII(S) 97.71, IncX1 98.66
BioSample no.	SAMN30076191	SAMN30076192	SAMN30076193	SAMN30076194
Genome Accession no.	JANKYP000000000	JANKYQ000000000	JANKYR000000000	JANKYS000000000

**Fig. 1:**
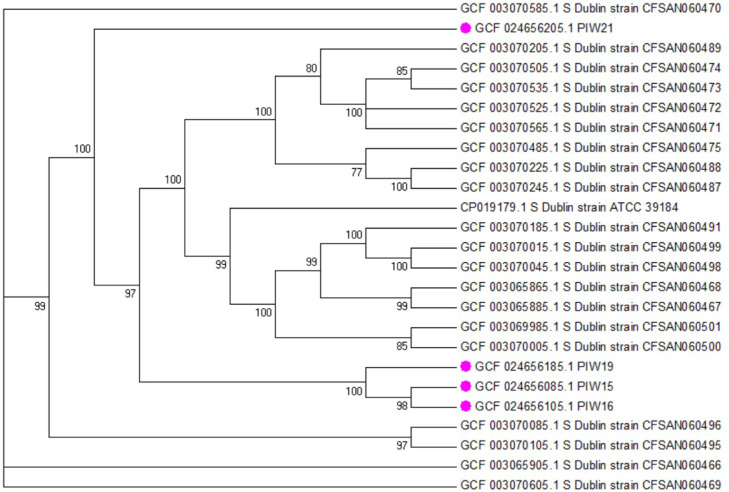
Phylogenetic tree of *S*. Dublin isolates (reported strains are marked with pink circles) based on cgSNP and reference strains downloaded from NCBI database. The bootstrap values are shown on branches. Tree visualization was made in MEGA 6.

**Fig. 2:**
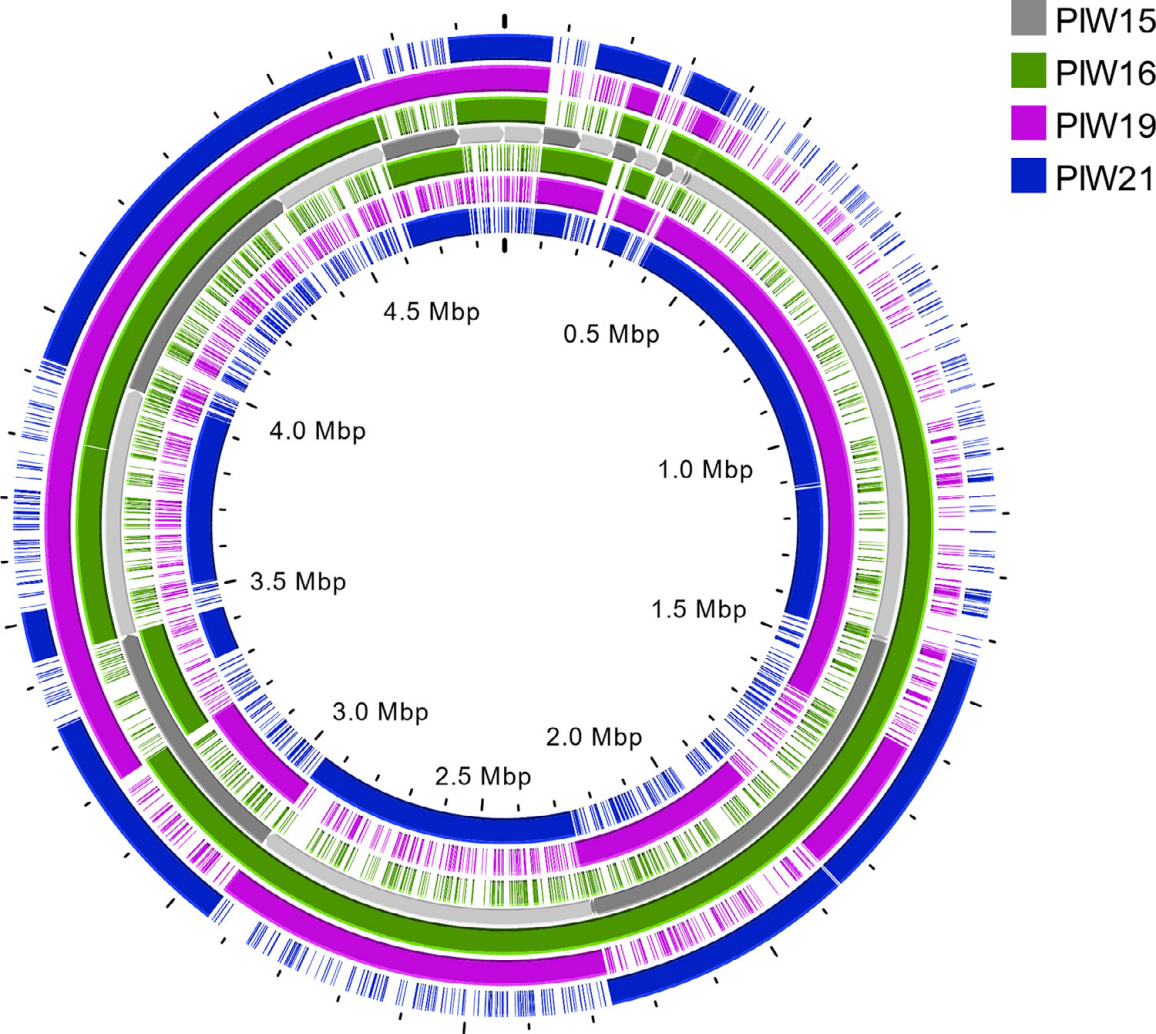
Circular map of the comparison of PIW15 (used as the reference), PIW16, PIW19, and PIW21. Outside from the PIW15 are CDSs on the forward strands, inside from PIW15 are CDSs on the reverse strands.

Core genome SNPs were used to create a phylogenetic tree (Fig. 1) of described isolates, the reference *S*. Dublin (ATCC 39184) and the another 20 genome sequences of cattle *S*. Dublin [6] available from NCBI database. The comparison of PIW15, PIW16, PIW19 and PIW21 sequences was also presented in the form of a circular map (Fig. 2).

## 3 Experimental Design, Materials and Methods

The lyophilized strains were rehydrated by adding 0,5 ml NaCl 0.85% Medium (bioMérieux) to each ampoule, the contents were gently mixed, then 10 μl of each suspensions were streaked onto Xylose Lysine Deoxycholate (XLD) medium and incubated overnight at 37±1 °C. Single colonies were passaged overnight at 37±1 °C onto nutrition agar and used for further analysis [7]. Strains identity was validated on the basis of biochemical reaction (VITEK System, bioMérieux), matrix-assisted laser desorption ionization-time of flight (MALDI-TOF) using the extraction method following the producer guidelines (Bruker Daltonik GmbH) and serologically according to the White-Kaufmann-Le Minor scheme. Isolation of genomic DNA was executed using Maxwell Rapid Sample Concentrator (RSC) cultured cells DNA Kit (Promega). Sequencing libraries were constructed using the Nextera XT sample preparation kit following the manufacturer's recommendations and evaluated by capillary electrophoresis (Fragment Analyzer). Whole genome sequencing was performed on the MiSeq platform (Illumina) with the MiSeq reagent kit (2 × 300-bp paired-end protocol, to 100 × depth of sequencing). Default parameters were used for all software unless otherwise specified. Raw paired-end reads were quality controlled using FastQC v0.11.5 [8] (https://www.bioinformatics.babraham.ac.uk/projects/fastqc/). Trimmomatic 0.36 [9] was used to trimmed and removaled adapter sequence with the following parameters: ILLUMINACLIP: 2:30:10, LEADING:3, TRAILING:3, SLIDINGWINDOW:4:15, MINLEN:36. To merge the trimmed reads the BBMerge from bbtools software suite was used (https://jgi.doe.gov/data-and-tools/software-tools/bbtools/bb-tools-user-guide/bbmerge-guide/) and assembled using SPAdes v3.9.0 [10] with the “-careful” flag. The genome statistics and annotation of the *Salmonella* strains were determined using the Quality Assessment Tool for Genome Assemblies (QUAST) [11] and NCBI Prokaryotic Genome Annotation Pipeline (PGAP) [12]. Bioinformatics tools from Center of Genomic Epidemiology (CGE) have been used to determine MLST type (MLST 2.0) and cgMLST (cgMLSTFinder 1.2) [13,14]. A phylogenetic tree was created in CSIPhylogeny and visualized in MEGA 6 [15]. The similarity of the sequences is shown on the circular map and generated in Proksee server (https://proksee.ca/).

## Ethics Statements

Not required.

## CRediT Author Statement

**Milena Skóra:** Investigation, Writing – Original Draft; **Renata Kwit**: Investigation, Writing – review & editing; **Magdalena Zając**: Conceptualization, Data curation, Formal analysis, Investigation, Methodology, Supervision, Writing – review & editing; **Marta Pietruk:** Writing – review & editing; **Magdalena Skarżyńska:** Writing – review & editing; **Ewelina Skrzypiec:** Writing – review & editing; **Katarzyna Tłuścik:** Writing – review & editing; **Anna Lalak:** Writing – review & editing; **Dariusz Wasyl:** Data curation, Writing – review & editing.

## Declaration of Competing Interest

The authors declare that they have no known competing financial interests or personal relationships that could have appeared to influence the work reported in this paper.

## Data Availability

Salmonella Dublin isolated from animal source Genome sequencing and assembly (Original data) (National Center for Biotechnology Information) Salmonella enterica subsp. enterica serovar Dublin str. ATCC 39184 chromosome, complete sequence (Reference data) (National Center for Biotechnology Information) GenomeTrakr Project: US Food and Drug Administration (Reference data) (National Center for Biotechnology Information)
